# LONG-TERM CARE NEEDS BY CHINESE ELDERLY AND POLICY PRIORITIES IN CHINA

**DOI:** 10.1093/geroni/igz038.916

**Published:** 2019-11-08

**Authors:** Juan Juan Sun, Haichao Wu

**Affiliations:** Institute of Gerontology School of Sociology and Population Studies, Renmin University of China, Beijing, Beijing, China (People’s Republic)

## Abstract

With the life expectancy in China continuing to increase, age-dependent chronic diseases are also likely to increase, as is the number of people with long-term care needs. This study evaluated the Long Term Care (LTC) needs of the Chinese older population and introduced related policy priorities. Using the 2014 and 2016 “China Longitudinal Aging Social Survey”, this study assessed the physical functions of older adults by measuring their ability to perform Activities of Daily Living independently, compared changes within the two years, and explored other related indicators including, Instrumental Activities of Daily Living, major chronic disease, and mental health conditions. The study also discussed the development of long-term care policies in China and highlighted the priorities of these policies.

